# Thyrotoxic Periodic Paralysis, an Unusual Presentation of Paralysis After Spinal Surgery: A Case Report

**DOI:** 10.7759/cureus.55822

**Published:** 2024-03-08

**Authors:** Ali Lattouf, Sohaib Alhamss, Amna Kiani, Naisargee Solanki, Nidal Younes

**Affiliations:** 1 Internal Medicine, Wayne State University, Detroit Medical Center, Detroit, USA; 2 Urology, Faculty of Medicine, Islamic University of Gaza, Gaza, PSE; 3 General Surgery, Faculty of Medicine, University of Jordan, Amman, JOR

**Keywords:** hyperthyroidism, hypokalemia, muscle weakness, periodic, thyrotoxicosis

## Abstract

Thyrotoxic periodic paralysis (TPP) is a rare complication of hyperthyroidism, seen predominantly in men of Asian origin. We report an unusual presentation of paralysis post-lumbar laminectomy surgery, associated with shortness of breath and arrhythmia. The patient was initially thought to have nerve compression as a complication of surgery but was found to have severe hypokalemia, which responded to intravenous potassium supplements. Additional tests identified suppressed thyroid stimulating hormone (TSH). The patient was diagnosed with thyrotoxic periodic paralysis (TPP), which was treated with oral potassium supplements and antithyroid drugs, followed by a total thyroidectomy. The report discusses the epidemiology, presentation, treatment, and complications of this rare condition.

## Introduction

Thyrotoxic periodic paralysis (TPP) is a rare complication of hyperthyroidism [1]. It is most commonly seen in Asian males between the second and fourth decade of life, with an incidence of 2% in patients with thyrotoxicosis [2,3]. Etiology includes emotional stress, strenuous exercise, a carbohydrate diet, and steroids that can precipitate an attack of TPP [1]. It is characterized by hypokalemia and paralysis, which usually affects the lower extremities [3]. The treatment of TPP includes potassium replacement, using non-selective beta-blockers to prevent the shift of potassium, and correcting the underlying hyperthyroid state [4].

## Case presentation

A 44-year-old male patient, not known to have any medical illness, presented with complete paralysis associated with shortness of breath and arrhythmia four hours after a lumbar laminectomy surgery. The patient denied any vomiting, change in bowel habits, palpitation, dysphagia, dysphonia, dyspnea, or heat intolerance. The remaining review of systems was negative. He was a smoker and denied the use of alcohol, recreational drugs, diuretics, or laxatives. There was no significant past illness or radiation exposure. The family history was unremarkable for TPP or any other thyroid diseases.

Physical examination revealed a normal-weight male who appeared conscious, alert, oriented to time, place, and person, and in no distress with a blood pressure of 123/87 mmHg, a pulse of 77 beats per minute, a respiratory rate of 15 breaths per minute, a temperature of 37.1 °C, and an oxygen saturation of 97% on room air. The head examination was negative for exophthalmos, lid lag, lid retraction, or temporal muscle wasting. The neck examination showed bilateral symmetrical diffuse thyroid enlargement with firm consistency and a lack of bruit. Cardiopulmonary and abdominal examinations were normal. The neurological examination was negative for fine tremors and positive for brisk reflexes.

Initial laboratory studies on presentation showed a serum potassium level of 1.22 meq/L (normal 3.5-5.3), a thyroid stimulating hormone (TSH) level of 0.0003 ulU/ml (normal 0.55-4.78), and a free thyroxine (T4) level of 22.4 pmol/L (normal 10-22). The electrocardiogram showed sinus tachycardia with a second-degree type 1 AV block. An initial diagnosis of hyperthyroid hypokalemic paralysis was made, and the patient was started on intravenous and oral potassium chloride (KCL) until his symptoms resolved.

Neck ultrasound showed a mildly enlarged homogeneous echo pattern of the thyroid gland with markedly increased vascularity and no dominant or discrete nodules. The right lobe measured 2.3x2x4.5 cm, and the left lobe measured 2.1x2x6 cm. A thyroid uptake scan confirmed the diagnosis of diffuse toxic goiter.

The patient was discharged home on carbimazole 20 mg daily with a diagnosis of TPP secondary to newly onset Graves’ disease. At the 12th week follow-up, the patient was found to be off the carbimazole, and the symptoms recurred on several occasions. He was then resumed on carbimazole 5 mg and potassium supplements and was planned for a total thyroidectomy. After the surgery, the patient had dramatic improvement with no episodes of muscle weakness, and his laboratory studies returned to normal levels.

## Discussion

Periodic paralysis (PP) is a type of muscle channelopathy group of diseases that are either hereditary with an autosomal dominant inheritance pattern called familial periodic paralysis (FPP) or acquired with the association of hyperthyroidism called thyrotoxicosis periodic paralysis (TPP) [5]. Occasionally, TPP is misdiagnosed with FPP due to the similarities. Familial periodic paralysis (FPP) is an autosomal dominant disorder caused by a defect in the gene coding for the L-type calcium channel 1-subunit on chromosome 1q31-32, whereas TPP usually has no family history, although the presence of the HLA DRw8 gene in certain ethnic populations plays a significant role in the susceptibility to TPP among those populations [1,6,7]. Most cases of TPP reported in individuals of Asian descent revealed muscle weakness or paralysis in the lower as opposed to upper extremities and proximal muscles more than distal, with typically preserved bowel and bladder functions [3,8].

The etiology of TPP is related to skeletal muscle ion channel defects causing intracellular shifts of potassium due to higher sodium-potassium adenosine triphosphatase (Na+/K+-ATPase) pump activity, and the increased pump activity can be explained by the release of catecholamine after exercise, the release of insulin after a rich carbohydrate diet, and the higher muscle-to-body ratio in men [3,9,10]. Diagnosis can be made based on the clinical presentation, lab results of potassium serum, triiodothyronine (T3), T4, and TSH, and ECG. A muscle biopsy may sometimes be warranted, showing atrophy of muscles, fatty infiltration, sarcolemmal nuclear proliferation, mitochondrial changes, and vacuolation, which can interfere with muscle contraction, leading to weakness [3,11,12]. Thyrotoxic periodic paralysis is treated with potassium supplements, nonspecific beta-blockers, and correcting the hyperthyroid state.

Early recognition of TPP is important to initiate appropriate treatment to avoid the risk of rebound hyperkalemia if a higher dose of potassium replacement is given [3,10]. Nonspecific beta blockers can terminate neuromuscular symptoms while reducing the intracellular shift of potassium [3,13]. Treating hyperthyroidism prevents attacks and can reverse muscle weakness [3,10].

## Conclusions

This is an unusual presentation of TPP following spinal surgery. Diagnosis should be made carefully due to the similarities with FPP and based on the clinical presentation associated with low potassium, low serum TSH, high serum T3 and T4, diffuse thyroid uptake scan, and abnormal ECG. During the treatment, serum potassium levels should be monitored due to the risk of rebound hyperkalemia. A total thyroidectomy can be offered as the definitive treatment for TPP.
